# Supplementary figures and images for: MicroRNA-26b protects against MASH development in mice and can be efficiently targeted with lipid nanoparticles
Source: eLife. 2025 Apr 22;13:RP97165. doi: 10.7554/eLife.97165 (PMC12014130; doi:10.7554/eLife.97165)

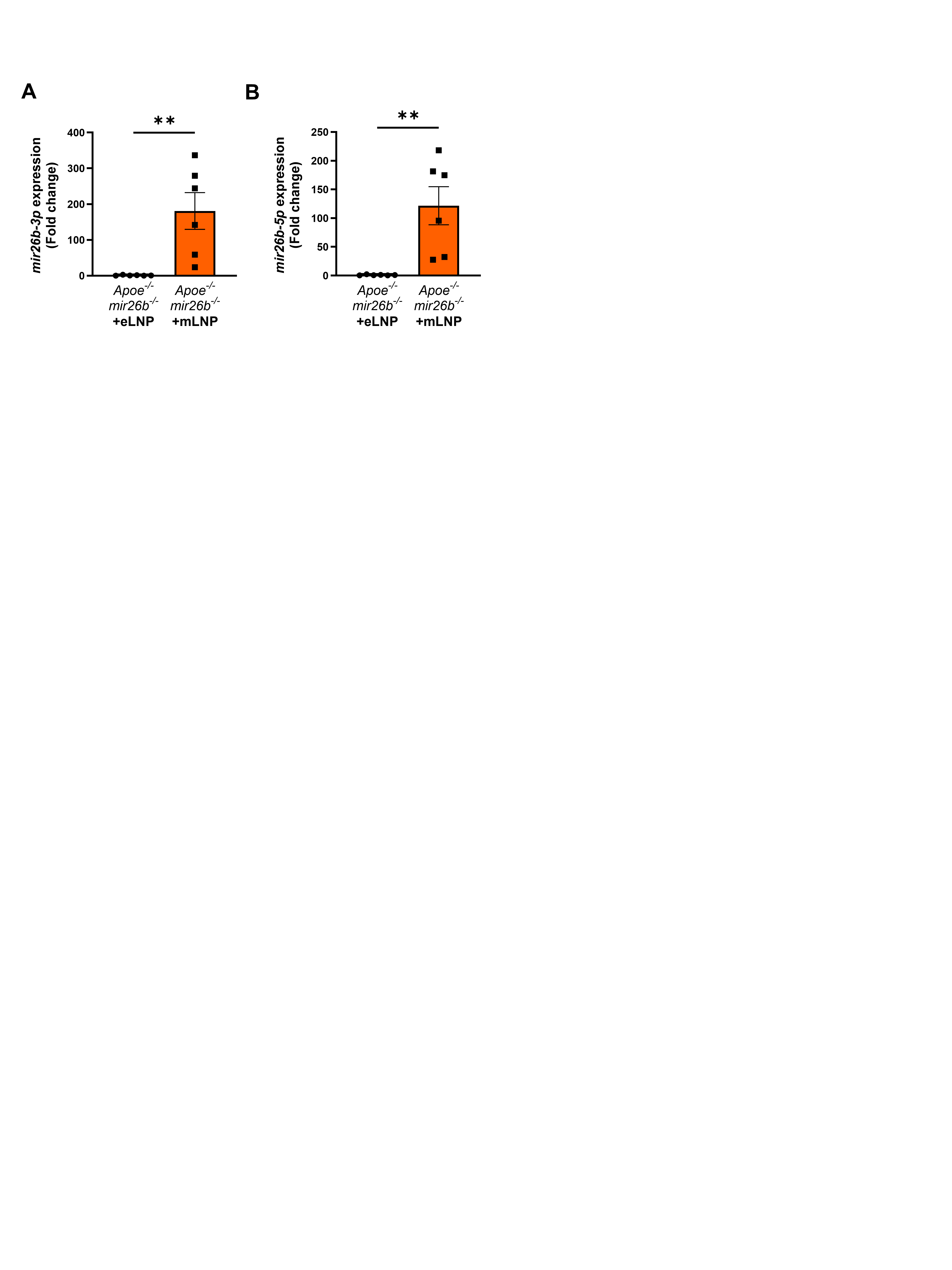

Supplement: Figure 6—source data 1. [file elife-97165-fig6-data1.tif]
